# *‘If I do 10–15 normal deliveries in a month I hardly ever sleep at home.’* A qualitative study of health providers’ reasons for high rates of caesarean deliveries in private sector maternity care in Delhi, India

**DOI:** 10.1186/s12884-018-2095-4

**Published:** 2018-12-03

**Authors:** Alison Peel, Abhishek Bhartia, Neil Spicer, Meenakshi Gautham

**Affiliations:** 1 London School of Hygiene and Tropical Medicine, London, UK; 2 Sitaram Bhartia Institute of Science and Research, New Delhi, India; 3 Department of Global Health and Development, Faculty of Public Health and Policy, London School of Hygiene and Tropical Medicine, London, UK

**Keywords:** Caesarean section rates, Private sector, Qualitative interviews, Delhi, India

## Abstract

**Background:**

Although the overall rate of caesarean deliveries in India remains low, rates are higher in private than in public facilities. In a household survey in Delhi, for instance, more than half of women delivering in private facilities reported a caesarean section. Evidence suggests that not all caesarean sections are clinically necessary and may even increase morbidity. We present providers’ perspectives of the reasons behind the high rates of caesarean births in private facilities, and possible solutions to counter the trend.

**Methods:**

Fourteen in-depth interviews were conducted with high-end private sector obstetricians and other allied providers in Delhi and its neighbouring cities, Gurgaon and Ghaziabad.

**Results:**

Respondents were of the common view that private sector caesarean rates were unreasonably high and perceived time and doctors’ convenience as the foremost reasons. Financial incentives had an indirect effect on decision-making. Obstetricians felt that they must maintain high patient loads to be commercially successful. Many alluded to their busy working lives, which made it challenging for them to monitor every delivery individually. Besides fearing for patient safety in these situations, they were fearful of legal action if anything went wrong. A lack of context specific guidelines and inadequate support from junior staff and nurses exacerbated these problems. Maternal demand also played a role, as the consumer-provider relationship in private healthcare incentivised obstetricians to fulfil patient demands for caesarean section. Suggested solutions included more support, from either well-trained midwives and junior staff or using a ‘shared practice’ model; guidelines introduced by an Indian body; increased regulation within the sector and public disclosure of providers’ caesarean rates.

**Conclusions:**

Commercial interests contribute indirectly to high caesarean rates, as solo obstetricians juggle the need to maintain high patient loads with inadequate support staff. Perceptions amongst providers and consumers of caesarean section as the ‘safe’ option have re-defined caesareans as the new ‘normal’, even for low-risk deliveries. At the policy level, guidelines and public disclosures, strong initiatives to develop professional midwifery, and increasing public awareness, could bring about a sustainable reduction in the present high rates.

## Background

Caesarean section can be a life-saving intervention during deliveries where the mother or baby is at significant risk of adverse outcome. Many high maternal mortality regions struggle to provide safe and timely caesarean sections in such circumstances [1], yet women worldwide are increasingly undergoing procedures for which there is little clinical justification [2]. These ‘unnecessary’ caesarean sections have been associated with increased maternal risk of severe morbidity and even mortality [3, 4]. High rates of caesarean sections also have economic repercussions on patients and their families, as financial costs for the procedure are higher than for vaginal birth and a longer period of hospitalization is necessary. This is especially true in low- and middle-income countries where medical care is often purchased ‘out-of-pocket’ [5]. It is widely accepted, including by the World Health Organization (WHO), that caesarean deliveries should only be undertaken where medically necessary [3, 4, 6, 7].

Caesarean section rates vary widely across the globe, ranging from as low as 1% in parts of Sub-Saharan Africa to 30% in the USA and 45% in Brazil [5]. Following debate over the most appropriate rates of caesarean sections at national and regional levels, the WHO issued a statement in 2015 recommending that every effort should be made to provide caesarean sections to women *in need,* rather than striving to achieve specific population-level rates [6]. At the facility-level, it is recommended the ‘Robson classification’ system be used as it allows for comparison of caesarean rates within and across different risk groups of women [6, 8].

A wide variety of explanations have been proposed for rising caesarean rates, ranging from medical and demographic factors to changing patient expectations and provider practices. Repeat caesareans are an important contributor to high rates, as previous caesarean delivery pre-empts need for the procedure in successive deliveries. The risk of morbidity increases with each procedure [9].

Studies in multiple settings have found high rates of caesarean sections associated with non-medical factors. Higher caesarean rates are consistently associated with delivery by private providers, and thus have often been linked to financial incentives [10–19]. Research in private facilities has also shown that both planned and unplanned caesarean sections, including some emergency ones, are more likely to occur on weekdays or during daylight hours [20–22]. It is possible that elective procedures account for many of these daytime caesareans, and that some emergency caesareans may be held over until day staff come on duty, rested and alert, to ensure better outcomes. However, these studies [20–22] also suggest that physician factors, including convenient timing of deliveries and desire for increased leisure time, are significant predictors of caesarean sections. Obstetricians’ fears of complaints and legal action have also been indicated as determinants of caesarean delivery [23–27], and it has been suggested that providers perceive fewer risks, both legal and medical, associated with caesarean versus vaginal deliveries [4, 26]. Another concept frequently cited in literature is the influence of patient-related factors, such as maternal request for caesarean section. Maternal demand could be motivated by a number of non-medical factors including fear of vaginal birth, need for control, and cultural acceptability of the procedure [28]. One study in the UK suggested that doctors perceived maternal request as the most important factor driving caesarean rates, although few actually reported receiving a large number of requests or performing caesareans on request themselves [26].

### The Indian context

India has made significant progress in improving maternal health, with the current maternal mortality ratio almost half of what it was in 2000 [29]. As mortality rates fall and increasing numbers of women gain access to formally trained maternity care providers, improving quality of care is becoming a priority for policy-makers. Indeed, some data suggest that quality of care is generally poor, though variable, in both the public and private healthcare sectors [30]. The private sector currently accounts for more than 70% of primary healthcare in India following rapid growth in recent decades [31]. This expansion has been accompanied by very little regulation or quality assurance, and insufficient standardisation of treatments, protocols and pricing. Private medical insurance is becoming increasingly common, although a large proportion of people still pay for care ‘out-of-pocket’ and are vulnerable to financial impoverishment if they require expensive surgeries or medication [32].

Private providers play a significant role in maternity care in India, accounting for 48% of institutional childbirths in urban areas and 24% in rural areas [31]. In Delhi, as in many other large cities in India, the vast majority of deliveries in the private sector are undertaken by obstetricians, who typically operate fee-for-service solo practices. There is very little practice of midwifery in urban hospitals and obstetricians often assume the role of the primary carer during deliveries, with limited support from other healthcare staff, especially midwives [33]. The size and structure of private sector facilities varies widely, from small nursing homes to large hospitals that are part of multi-national corporate chains [14].

Although the overall rate of caesarean deliveries in India is around 17%, rates have risen rapidly over the last ten years from 8.5% in 2005–06 to 17.2% in 2015–16 [34], driven particularly by increases in the private sector and in urban areas. Data collected over 2 years in the city of Chennai in south India indicated a caesarean rate of 47% in private healthcare [18]. Other studies have reported that women in the states of Kerala, Goa, Andhra Pradesh, Bihar, Gujarat, Karnataka, Punjab, Uttar Pradesh, Delhi, Maharashtra, and Tamil Nadu are far more likely to deliver by caesarean if they receive private care [11, 13, 15]. A community-based household survey indicated that 54% of women delivering in the private sector in Delhi underwent caesarean section, compared with a 24% rate in the public sector [14]. Although rates were not classified according to women’s risk-status, this figure appears to indicate overuse of the procedure.

A small number of studies have presented data that begin to explain *why* caesarean section rates in the Indian private sector are higher than in the public sector. An analysis of supplier-induced demand in Madhya Pradesh and Gujarat [35] found that direct economic incentives led to increases in caesarean rates whilst financial disincentives led to decreasing rates. This suggests that private obstetricians induce maternal demand for caesarean section due to profit-making motives. Other studies have identified a positive correlation between maternal educational level and likelihood of caesarean delivery in private facilities, suggesting that maternal demand also plays a role, particularly amongst women with a higher level of education [15, 17]. Despite these findings, one of the studies [15] suggests that rising rates of caesarean delivery are more a result of supply factors (relating to the obstetrician) than maternal demand.

A number of studies reporting high rates of caesarean sections in the private healthcare sector in India have hypothesized that these reflect financial incentives, time pressures, the custom of solo practice, fear of litigation, maternal request, and use of intensive foetal monitoring [13, 14, 18]. However, it was not within the remit of the studies to test these hypotheses, and so the evidence remains lacking. No studies have explored providers’ perspectives of the reasons for high caesarean rates in their private practices in India. The small quantity of data generated in other settings, especially high-income countries, may not be applicable to the Indian private sector. Our study sought to explore the perspectives of private healthcare professionals involved in maternity care in Delhi on reasons for high caesarean section rates, and solutions to reverse the trend of high rates in private maternity care in Delhi. The study is among the first to explore provider-side perspectives on this issue in the Indian setting.

## Methods

### Study setting

The study was located in Delhi and its neighbouring suburbs of Gurgaon and Ghaziabad, all in the National Capital Region (NCR) of India. Maternity care is delivered by a wide range of private and public facilities that vary greatly in size, structure, and staffing arrangements in these three settings. There are more than 560 private hospitals and nursing homes in Delhi alone [36]. Women of high socio-economic status are more likely to pay for private maternity care, with more than 80% attending private facilities. A substantial proportion of middle and low socio-economic status women (40 and 16%, respectively) also opt for private maternity care. Caesarean sections are more common in private facilities, where the rate is 54% compared with 24% in the public sector [14]. Our study focused on private maternity care providers in this setting. We collaborated with a private medical hospital and research institute based in South Delhi.

### Study design

Fourteen in-depth qualitative interviews were conducted during July and August 2015. We approached key informants who could offer insights from the perspective of a private sector maternity care provider. To get an all-round providers’ perspective, we purposively selected practicing obstetricians and a small number of other healthcare professionals with involvement in maternity care - two paediatricians, the manager of a private maternity hospital and a ‘doula’ or birth companion, who is professionally trained to provide physical, emotional and educational support to the mother. This gave us the opportunity to compare and contrast the perspectives of obstetricians with those of other professionals with good knowledge of the sector.

The majority of study participants worked in South Delhi, with an additional participant working in East Delhi and two in other cities in the NCR, Gurgaon and Ghaziabad. As the sector is highly diverse, we selected providers from secondary and tertiary facilities of different sizes, with capacity for performing caesarean sections: small hospitals or nursing homes (< 50 beds), medium-sized hospitals (50–150 beds), and a large corporate hospital (> 150 beds). These included a mix of multi-specialty and super specialty private hospitals where a normal delivery can cost upwards of INR 40,000 (US$ 615), and a caesarean section in a super luxury room can be priced as high as INR 12,00,000 (US$ 18,461). These were amongst the more high-end private hospitals in the NCR, serving the wealthiest socio-economic sections of the population, who would typically seek only private sector maternity care.

With the help of our collaborating research institute, we drew up an initial list of 19 potential respondents and contacted them for interviews. Ten agreed to an interview during the period that the primary researcher was available, and with the help of these respondents, we identified and interviewed an additional four who agreed.

The interviews were conducted using an interview guide that explored participants’ professional status and background, perceptions of current caesarean section rates in the private sector, awareness of guidelines regarding rates, reasons they attributed to high rates, and their suggested solutions for reducing rates.

Confidentiality was maintained by conducting the interviews in private rooms at participants’ workplaces or homes. Interviews were audio-recorded where permission was given. In the case of three participants where permission was not given, detailed hand-written notes were taken. All interviews were carried out in English by a single interviewer.

### Analysis

All audio-recorded interviews were transcribed. Transcripts and interview notes were coded manually using a coding frame developed both from concepts that emerged in the data and those found in the existing literature, for example financial incentives. Data were organised into matrices for each major topic to help systematize analysis, identify patterns in the data, and compare participants. A final list of key themes and sub-themes emerging from interviews was identified.

## Results

### Perceptions of caesarean rates in Delhi private sector

Most respondents were of the view that caesarean section rates in the private maternity homes they visited were unjustifiably high and that a substantial proportion of procedures were performed without clear medical need. Two obstetricians believed that rates could be as high as 90% in the different hospitals where they practiced. Others cited rates of 15–50% in their facilities. Most respondents acknowledged that, as high caesarean section rates were rarely discussed within the healthcare community, practitioners were unlikely to know precise facility-level or even personal rates and little self-auditing occurred in private hospitals.

“It’s not part of the Indian medical environment right now to really critically analyse their outcomes and compare it to standards.” (Hospital chief executive)

### Reasons for high rates of caesarean sections

Three groups of themes emerged in our analysis of participants’ explanations for high caesarean rates in the Delhi and NCR private sector: provider-related, system-related, and patient-related factors. These are presented in the following sections.

#### Provider-related factors

##### Personal convenience

The majority of respondents said that providers’ convenience, in terms of time spent and timing of deliveries, was the most important consideration for doctors. Vaginal deliveries could involve more than 12 h of labour and occur at inconvenient times, particularly during the night. Caesareans allowed doctors to exercise control over the duration and timing of delivery so that they had more time for personal and professional activities.

“One normal delivery costs me at least a night, sometimes 2 nights. If I do 10-15 normal deliveries in a month I hardly ever sleep at home. If I do 15 caesareans I’m not home late for coffee.” (Private sector obstetrician)

##### Concerns about decision-making

Many respondents spoke about the pressure of an obstetrician’s demanding workload, which meant that doctors were unable to commit their full attention to every delivery because they were pre-occupied by other concerns or a lack of sleep. This could affect decision-making regarding deliveries.

“Supposing one is awake the whole night for 2 days, the third night one will have a tendency to take weak decisions.” (Private sector obstetrician)

Another common theme was the pressure of having sole responsibility for a delivery (due to the norm of working alone), and having to make difficult, subjective decisions about the safest option for a patient.

“Decision-making is very tough. For caesarean anybody can make decision, but how long you have to wait for a normal delivery is very difficult to assess”. (Private sector obstetrician)

Some spoke about fearing for the safety of patients and their babies during deliveries. This fear could lead them to decide in favour of a caesarean section earlier than necessary.

“Whatever I was trained in just goes away…suddenly I think I have to give her a live baby” (Private sector obstetrician)

Fear of legal action by patients emerged as another important challenge to doctors’ decision-making. Respondents explained that, according to the existing laws, if anything goes wrong the sole legal responsibility lies with the obstetrician. Therefore, they considered caesarean as the safer option for avoiding litigation, as one described it, a caesarean was *“a more comfortable and assured path...” (*Private sector obstetrician*).*

Some respondents suggested that use of technologies such as ‘cardiotocography’ machines for continuous foetal heart rate monitoring could also be increasing the frequency of decisions for caesarean deliveries. They described doctors panicking or becoming *“hyper”* when they saw decelerations in foetal heartrate, leading them to perform a caesarean section even though the labour may have progressed normally.

##### Training and continuing medical education

Some interviewees explained that waiting for normal labour to progress might seem counterintuitive to doctors because of the training they have received. They argued that doctors are trained for pathological events and therefore they are predisposed to intervene, even during a physiologically normal birth.

“Doctors are not trained for sitting and watching and waiting, they are trained to intervene…they are people who need to jump in when something is going wrong” (Private sector paediatrician)

Aside from this, the providers interviewed thought that the initial training for obstetrics was adequate, although some expressed concern about obstetricians not updating themselves on current knowledge later in their careers. Although interviewees stated that continuing medical education was available for obstetricians, they indicated that many would not attend due to heavy workloads, or a perceived lack of need for additional training.

“Most of us don’t do trainings, we just run our own clinics and the basic idea is the financial situation is fine, and if you’re sensible enough, then you don’t do any harm to your patients” (Private sector obstetrician)

Respondents believed that, due to a lack of continuing medical education, some obstetricians were unaware of current evidence-based guidelines and performed unnecessary caesareans as a result.

Non-compliance with guidelines was also an issue. Many obstetricians reported working in hospitals where a set of standard guidelines were available. However, one protested that there is little compulsion for visiting consultants to follow guidelines.

“You cannot enforce the guidelines on them… if we force them they will not bring the patient to the hospital, so hospital will lose those number of clients.” (Private sector obstetrician)

As we report further on, respondents also expressed concerns over a lack of guidelines that were appropriate to the setting.

In addition, it emerged that many obstetricians associated little risk with caesarean deliveries. Respondents spoke about caesareans having become routine procedures, with doctors thinking of them as *“no big deal”.* Nevertheless, they also described adverse consequences associated with medically unindicated caesarean sections and believed that all obstetricians would be aware of these from their training. One respondent attempted to explain the disparity between these viewpoints:

“We all know what we’re doing, but of course the mind is between knowing and using that knowledge” (Professor of obstetrics).

Some respondents reasoned that a vaginal delivery may appear more risky to obstetricians as there were more ‘unknowns’ compared with a caesarean section.

##### Commercial interests

A few respondents mentioned financial incentives as a reason for high caesarean section rates in the private sector. However, they placed less weight on this than the other reasons. This could be because they felt uncomfortable admitting to monetary incentives in interviews, or because the financial benefits of caesarean deliveries affected them only indirectly.

Many respondents referred to hospitals, rather than individual doctors, being financially motivated. Several described how, in their experience, corporate hospitals were often run in a way where doctors were encouraged to take on as many patients as possible in order to generate revenue.

“[Hospitals] see the business that you are doing. They will not see how many caesareans or normal deliveries you’re doing…the doctor is under pressure to have more patients because that’s her power, her importance in the institution is what she brings to the hospital.” (Private sector obstetrician)

It was also reported that patients paid more for caesarean than vaginal delivery, due to additional charges for operating theatre rent, anaesthetists, and other necessary arrangements. Some suggested that these dis-incentivised facilities to decrease caesarean rates.

“Hospitals earn about 30-50% more revenue as the result of a caesarean delivery compared to a normal delivery. And almost all of the additional revenue is additional margin. So, hospitals have a clear financial incentive to not mind a high caesarean section rate.” (Hospital chief executive)

In contrast, some other interviewees were insistent that commercial incentives played no part in doctors’ decisions to perform caesarean sections.

“No no no no no! ...the charges [fees charged by doctors] for caesarean and normal delivery are almost at one. So that is never a reason.” (Private sector obstetrician)

This assertion that doctors’ fee for both modes of delivery were the same or very similar was reiterated by several respondents. Many emphasised that obstetricians *“don’t do it for money but to save time”.*

“An obstetrician would rather do a caesarean and go and save time, maximise the benefit of the time spent to do more deliveries, see more OPD patients.” (Private sector paediatrician)

Even those who believed that financial incentives played a role stated that time was a more important factor. However, this was put into perspective by one obstetrician who pointed out that by attending to more patients in less time doctors could take on more clients and thus generate more revenue.

Furthermore, a number of other respondents indicated that the commercial nature of the private health sector’s commercial nature prevents providers from lowering their caesarean rates. Doctors and hospital executives are compelled to maximise the number of patients they receive as it directly determines their income.

“If you want to do always what is right for the patient but you reward clinicians on volumes there’s a misalignment.” (Hospital chief executive)

As obstetricians were unlikely to turn patients away even if their workload was high, there was always great pressure on their time. This was especially likely for doctors visiting multiple hospitals, as one respondent described of an obstetrician she knew:

“She did clinics and had women waiting in labour at all of the hospitals. She was getting calls continuously. It’s that sort of life, very hectic. That will increase caesarean rates.” (Private sector paediatrician)

The norm of solo practice in the sector exacerbated this problem, as obstetricians could not rely on other doctors to assist them when they were short of time, unlike in a ‘shared’ practice.

#### System-related factors

Some interviewees believed that the structure of the private sector could be partially to blame for high caesarean rates. They emphasised lack of regulation as an important systemic factor. There were no government requirements for reporting on maternity care in the private sector, and some believed that this could be a reason for high rates.

“If I’m an obstetrician with a 70% caesarean rate I can go on that way for the next 30 years. Nobody will find out.” (Hospital chief executive)

Many respondents believed that obstetricians would be less likely to perform unnecessary caesareans if they knew their practice was being monitored.

Some respondents also pointed to the lack of clinical guidelines for making decisions about caesarean sections issued by an Indian body. Indeed, several obstetricians reported using guidelines from other countries and some expressed concern about their use in an Indian setting. Others mentioned that the Federation of Obstetric and Gynaecological Societies of India issues a limited number of guidelines relating to caesarean sections, but these were not comprehensive and awareness of them was low amongst obstetricians.

Poor support systems were also cited as a cause of high caesarean rates. Respondents said that the standards of support from junior and nursing staff in the private sector varied greatly.

“The nurses are not trained in midwifery, they are not taking part in the antenatal period…I think they are underutilised, which is a big problem” (Private sector obstetrician)

One obstetrician explained that *“...as a result* [of poor support]*, all of the decision-making responsibility flows straight up to the consultant” (private sector obstetrician),* highlighting the additional pressure on doctors caused by these inadequate support systems.

#### Patient-related factors

Respondents reported that caesareans frequently occur because of patient demand, sometimes scheduled beforehand, but often requested during the process of labour. Many expressed that caesarean sections were perceived as the ‘normal’ mode of delivery amongst women.

“Caesarean has become the new normal. Chances are my friends, my mum, more people that I know have had caesarean deliveries rather than normal deliveries.” (Hospital chief executive)

Respondents perceived that women viewed caesarean as an *“easy way out”* of the pain of labour, and spoke about women hearing accounts of traumatic experiences, through either word of mouth or the media, which led them to request caesarean deliveries. A couple of respondents also observed a link between fear of labour and women’s bodies, saying that this led to the body not responding well to natural birth, so that a caesarean was necessary. Other patient-related factors included the convenience of a *“short-cut”* delivery and desire to schedule deliveries on auspicious dates.

Some respondents expressed concern about the sources from which patients obtained their information on deliveries, which they perceived to be primarily peer groups and family. Several stated that women did not fully understand the risks of a caesarean delivery, or that women perceived caesarean to be a safer choice than normal delivery. Many thought that counselling of patients by doctors, where the relative risks and benefits of delivery methods were explained, was often insufficient or entirely lacking.

Obstetricians often agreed to patients’ demands and performed caesareans even though they were not medically necessary. This was attributed to the time and effort required to change a patient’s mind.

“The easiest approach is that I do what the patient is saying…the more I try to explain the more time is used…I might as well give her a date for caesarean in 5 minutes” (Private sector obstetrician)

Others spoke about patients who insisted on a caesarean delivery in spite of counselling, and felt that they could not *“force”* a woman to have a natural delivery. Some respondents said that patients’ families put pressure on doctors to intervene during labour. Consumer-provider type relationships in the private sector implied that obstetricians were willing to satisfy patients’ demands for caesarean sections. If they refused to perform a procedure the patient could easily go to a doctor who was more willing, and this would result in loss of patients. *“It’s a demand and supply kind of thing”* said one private sector obstetrician, emphasising that private sector clients have more power to make demands than in the public sector because they are paying customers.

In addition to ideas about patient’s expectations and choices, interviewees spoke about lifestyle changes, such as women having babies later in life or being more sedentary and overweight, which were related to the higher socio-economic status of patients using the private sector. Previous caesareans were another reason, as women were likely to require a caesarean section if they had had one previously, due to the increased risks of complications. One interviewee stated that vaginal birth after caesarean was *“almost zero”* in the private sector in Delhi.

#### Possible solutions to high caesarean rates

The most frequently mentioned solution was improved counselling by doctors, in order to reduce patient demand for caesareans.

“Shaping women’s attitudes and behaviour is a very important aspect of this challenge if we actually want to solve the caesarean problem.” (Hospital chief executive)

Many respondents called for better education of pregnant women about the vaginal delivery process, the risks associated with caesarean sections, and maximising the chances of a natural delivery. This would help women cope with labour pains and could be imparted during existing sessions, such as antenatal workshops.

Acknowledging the present lack of guidelines, several respondents suggested that standardised evidence-based guidelines, issued by an Indian body and tailored to the Indian setting, would be effective in reducing caesarean rates. They expressed that guidelines should be introduced at the institutional level at the least, and that doctors should be encouraged to follow them. One obstetrician, who managed the mother and child unit of a hospital, stated that introducing standard guidelines had been the most effective method for lowering numbers of caesarean deliveries in their facility.

A number of respondents believed that regulation would be important for enforcing these guidelines. Some suggested the possibility of a higher-level regulatory body auditing caesarean rates and imposing penalties where unnecessary procedures were being performed. Others thought that promoting transparency could begin to tackle the problem.

“If, as part of a professional code of conduct, I was required to disclose it [my caesarean rate], now suddenly women would know…there would be an incentive for everyone in the system to start rectifying the situation.” (Hospital chief executive)

Another solution was to improve support for obstetricians from other medical staff. Respondents emphasised that nurses should receive training to provide more *‘*hands-on’ support during labour. Some respondents suggested an expanded role for midwives as the primary carers in low-risk deliveries. A counter argument was that patients in private hospitals might not feel comfortable if they were attended by a midwife rather than a doctor. Therefore, an additional suggestion for improving support was the idea of *‘*shared practice’, where obstetricians could rely on each other for help with decision-making and performing deliveries.

## Discussion

The present study identifies a number of important factors that may be driving high rates of caesarean sections in the private healthcare sector in Delhi and its neighbourhood. The rates reported by some respondents for facilities where they practiced varied from 15 to 50%, and a small number reported rates as high as 90%. As self- or facility-level auditing of caesarean sections was not commonly practiced in these facilities, these rates may be considered as estimates rather than precise figures. Even so, the figure of 50% is comparable to rates reported by two household surveys in Delhi: the National Family Health Survey-4 [34] that reported 43% caesarean births in the private sector and another household survey conducted at a similar time [14] that found 54% caesarean section births in the private sector. The higher rate of 90% reported by a small number of our respondents could be more facility specific and indicative of the substantial variation likely to exist across facilities. Similar variation can be seen in reported rates ranging from 50 to 99% in a study of private health facilities providing delivery care in the state of Uttar Pradesh, which borders Delhi [37].

The respondents we interviewed were generally of the opinion that unnecessary use of caesarean sections was an important issue in this sector. Key contributors were identified as: obstetrician convenience and time pressures, particularly owing to the high prevalence of solo obstetric practice; a perception of caesarean deliveries as the ‘safe’ option, both in terms of maternal health and protection from litigation; and financial pressures associated with running a successful clinic, or working for a commercial hospital. Other important reasons for high rates of caesarean deliveries included system-related factors, especially the lack of comprehensive guidelines tailored to the Indian setting, and lack of well-trained support staff such as midwives, as well as patient-related factors such as maternal and family related fears and demands. Interestingly, none of our respondents mentioned emergency referrals from smaller, more poorly-equipped private facilities or from government facilities as an important reason for the high caesarean rates. In a recent study in Uttar Pradesh [37], emergency referrals for caesarean sections were commonly reported by secondary and tertiary private facilities located on the outskirts of big cities and in smaller urban centres. Our study sample was quite different from these types of peri-urban facilities. Our respondents were all located within a metropolis, where most delivery facilities, private as well as public, have the capacity for performing caesarean sections and do not need to refer frequently. Moreover, the clientele at these facilities belonged to the wealthiest socio-economic groups who could afford pregnancy and delivery care at the best equipped private facilities from the very beginning.

Although respondents gave a variety of reasons for high caesarean rates, many of these can be retraced to the commercial nature of private sector practice that incentivises growing practices even to the point that time pressures interfere with the ability to provide quality care. Obstetricians must maintain their patient loads in order to run commercially viable practices, leading to immense pressures on their time. They must protect their patients from any adverse outcomes, and themselves from litigation. It is not possible to monitor every delivery to its normal conclusion, and so obstetricians may opt for caesarean deliveries in order to ensure patient safety and sufficient time to attend to all of their patients. As a result, caesarean section has come to be considered as the ‘safe’ option, in spite of the associated risks. A culture amongst obstetricians of performing caesarean sections, where ‘caesarean is seen as the new normal’, reinforces this perception and may prevent providers from recognising high caesarean rates as abnormal or harmful. Furthermore, inadequate support systems, including very limited practice of midwifery in India, increase time pressures on obstetricians and the difficulty of decision-making regarding deliveries.

Maternal and family requests for caesarean section were also identified as a driver of caesarean rates. Providers perceived a culture where caesarean sections are considered an ‘easy’ option among women using private sector facilities. The apparent frequency with which obstetricians fulfil patients’ requests for caesarean section reflects the provider-consumer type exchange between doctors and patients due to the commercial nature of their relationship. Doctors may feel obliged to fulfil patients’ demands or refrain from taking a hard line during patient counselling in order to avoid losing that patient. Our data suggest that some women who request caesarean deliveries receive insufficient counselling regarding the associated risks, likely due to the pressures on obstetricians’ time. Respondents did, however, identify physical factors, such as women postponing pregnancy until later in life or being overweight because they were leading more sedentary lifestyles, as a cause of rising caesarean rates, particularly amongst the high socio-economic status women who use private healthcare.

A lack of guidelines and regulation regarding caesarean sections amplify the effects of rising caesarean section rates, as obstetricians and institutions are not held accountable for their rates. The Indian government’s most recent guidelines for dealing with obstetric complications were issued in 2005. These detail the complications for which a caesarean section may be necessary, but no thresholds for caesarean section are given and few associated risks are mentioned [38].

The solutions that respondents offered for reducing caesarean section rates reinforce these explanations. They suggested that the level of support from junior and nursing/midwifery staff be improved, by either expanding the roles of support staff or encouraging small groups of obstetricians to work in ‘shared practice’, providing professional support to one another where necessary. Expanding the role of midwives to be the primary carers during normal deliveries, with support from obstetricians only when complications occur, could significantly reduce the time pressures on doctors and thus help avoid unnecessary caesareans. Unfortunately, midwifery remains an under-developed and under-recognised profession in India [33], with neither legislative support nor training standards for independent midwifery. The currently available training in midwifery, which is limited to a few months and combined with more general nursing training, has been found inadequate for preparing confident and competent midwives [39]. Greater policy support is required to promote midwifery in India, although this is likely to be met with initial resistance from providers and patients who are accustomed to doctors being present throughout the delivery. Other suggestions were that comprehensive caesarean section guidelines be made available from an Indian medical body, and that individual and institutional caesarean section rates undergo auditing and regulation. Some form of peer review within the sector, or regulation by an external body, could help to improve obstetricians’ awareness of their own rates and incentivise them to only perform procedures in cases where they are truly necessary.

The present study adds depth to the current understanding of high caesarean rates in the Indian private sector. Our findings support results from previous studies both in Indian and non-Indian settings, which emphasise obstetrician time and convenience factors [20–22], fear of litigation [23–27], financial incentives [10–19], perceptions of low risk [4, 26], and maternal demand [26, 28] as important reasons for high caesarean section rates. In addition, the present study identifies some previously undocumented factors that may contribute to high caesarean rates in the Indian setting: a lack of appropriate guidelines on caesarean sections, and the culture of private sector obstetricians working in solo practice, often with insufficient support systems, particularly well-trained midwives. Furthermore, our analysis highlights the ‘missing’ link between caesarean sections and obstetricians’ high practice volumes as a result of financial incentives in the private sector.

### Limitations

The small sample size of this study, due to resource constraints and the difficulties in accessing busy obstetricians and other maternity healthcare providers, limits its generalisability. Our sample was also mostly limited to the higher end obstetricians and multi-speciality/super-specialty private hospitals that cater to wealthier, urban clients that can afford to pay a high fee for institutional deliveries in big city hospitals. The situation in smaller, less expensive private hospitals in peri urban and rural areas is likely to be quite different and needs to be explored through other studies. Another limitation was that we could not access any documented data on caesarean rates from the facilities and, therefore, the rates we present here are reported estimates. However, it is unlikely that this information would have been available in many cases, as the respondents themselves reported that little self-auditing occurred amongst providers. We have also shown that the estimates are comparable to rates reported in the published literature. Finally, we did not interview any patients in this study, as our focus was on providers’ perceptions. Patients’ perceptions could be very different, and especially useful for understanding the extent to which maternal requests play a role in this setting. Nonetheless, the providers’ perceptions reported in this article are a valuable addition to the literature. We have reached out to an important and challenging group of providers and gathered rich and in-depth insights on a sensitive topic that will be useful for exploring caesarean reduction strategies, and designing further research around this important topic.

## Conclusions

A complex relationship exists between high caesarean rates and the commercial nature of the private sector. Although there may be no direct link between providers’ decisions for a caesarean section and financial gain, obstetric practice in the private sector is dependent on maintaining high patient loads. This can lead to doctors taking on more patients than they can feasibly manage, and opting for caesarean deliveries in order to ensure patient safety, as well as protection from litigation.

Reducing high rates of caesarean deliveries in the Delhi private sector will depend on the introduction of comprehensive caesarean section guidelines, including indications and thresholds for which the procedure should be performed, and public disclosure of the caesarean rate for individual obstetricians and hospitals. However, regulations and guidelines may be insufficient without a parallel strengthening of professional midwifery support for obstetricians, before, during and after childbirth, and improved patient counselling and awareness. As India’s most prominent obstetric body, the Federation of Obstetrics and Gynaecological Societies of India could play an important role in steering a comprehensive and sustainable caesarean reduction strategy in the higher end private sector in India.

## Abbreviations

NCR: National Capital Region
UK: United Kingdom
USA: United States of America
WHO: World Health Organization
